# Immune Regulatory 1 Cells: A Novel and Potent Subset of Human T Regulatory Cells

**DOI:** 10.3389/fimmu.2021.790775

**Published:** 2022-02-08

**Authors:** Nicolas Krause, Jörg Mengwasser, Elpida Phithak, Francisca Beato, Marc Appis, Edgar Louis Milford, Johan Pratschke, Igor Sauer, Anja Kuehl, Arndt Vogel, Michael Goodyear, Linda Hammerich, Frank Tacke, Johanna Faith Haas, Tobias Müller, Nalan Utku

**Affiliations:** ^1^ Institute for Medical Immunology, Charité – Universitätsmedizin Berlin, Berlin, Germany; ^2^ Department of Hepatology and Gastroenterology, Universitätsmedizin Berlin, Berlin, Germany; ^3^ Department of Surgery, Charité – Universitätsmedizin Berlin, Berlin, Germany; ^4^ Department of Gastroenterology, Moffit Cancer Center, Tampa, FL, United States; ^5^ Department of Biochemistry, Freie Universität, Berlin, Germany; ^6^ Department of Medicine, Renal Division, Brigham and Women’s Hospital, Harvard Medical School, Boston, MA, United States; ^7^ Department of Gastroenterology, Hepatology and Endocrinology, Hannover Medical School, Hannover, Germany; ^8^ Department of Medicine, Dalhousie University, Halifax, NS, Canada; ^9^ Department of Hepatology and Gastroenterology, Charite, Berlin, Germany; ^10^ Sachs Incubator for Translational Medicine, Sächsische Inkubator für Klinische Translation (SIKT), University of Leipzig, Leipzig, Germany

**Keywords:** T regulatory cells, IR1 cells, TIRC7, autoimmunity, immune regulatory Cell 1

## Abstract

A subset of T regulatory cells (Tregs), identified by TIRC7 (T cell immune response cDNA 7) expression is designated as Immune Regulatory 1 Cells (IR1 cells). TIRC7 is an immune checkpoint inhibitor, co-localized with the T- cell receptor, HLA-DR and CTLA-4 during T-cell activation, which delivers regulatory signals *via* binding to its ligand, HLA-DR *α*2 domain. IR1 cells express FOXP3, and multiple other markers associated with immune suppression. They constitute as much as 10% of Tregs. IR1 cells strongly inhibit proliferation in mixed lymphocyte reactions, where they express high levels of IL-10. *Ex vivo* expansion of Tregs over 2 weeks in the presence of an agonist TIRC7 antibody disproportionately expands the IR1 Treg subset, while maintaining high expression of suppressive markers including CD39, IL-10, LAP and GARP. *Ex vivo* expanded IR1 cells are a potent, homogeneous, stable set of suppressor Tregs with the potential to modulate immune dysregulation. The characteristics of IR1 cells suggest a therapeutic advantage over polyclonal Tregs for therapeutic interventions. Early restoration of immune homeostasis using IR1 cells has the potential to fundamentally alter the natural history of conditions characterized by abnormalities in the T regulatory cell compartment.

## Introduction

Thymus-derived T lymphocytes form the basis of cellular immunity in humans. T cells perform a wide range of functions, including the initiation and maintenance of immune response and memory. CD4+ regulatory T cells play an essential role in immunological homeostasis (1, 2). Treg immunosuppressive properties have generated interest for their therapeutic potential in a wide range of clinical indications. Expression of CD4 and CD25 were early indicators of a subset of regulatory cells able to transfer tolerance in autoimmune conditions. Expression of the transcription factor FOXP3, encoded by an X-linked gene (3), and low expression of CD127 are also markers of T suppressive function (4). Tregs, defined by marker phenotype CD4+CD25+CD127loFOXP3+, make up less than 10% of human CD4+ cells (1) and are heterogeneous. It is important to identify functional subgroups to optimize therapeutic Treg cell interventions (5, 6). To this end, we have investigated the association of key measures of T-regulatory cell suppressive activity, and the presence of the immune surface marker TIRC7, in human T-regulatory cells.

We have previously identified TIRC7 (T-cell immune response cDNA 7) (7, 8), a multimembrane spanning protein, co-localized with the T cell receptor, HLA-DR and CTLA-4 (9, 10), which is induced within hours of T cell activation (11). TIRC7, also referred to as TCIRG1 (T cell immune regulator 1), and its splice variant OC116, expressed in bone tissues, share the same chromosomal location at 11q13.4-q13.5 (12). TIRC7 induction is largely restricted to activated lymphocytes and lymphoid tissue (13). The natural ligand of TIRC7 is the HLA-DRα2 domain (14). TIRC7 disruption in mice leads to a hyperproliferative phenotype of T and B cells with an increase in the effector memory cell population (8). Previous work has shown that activation of TIRC7 by soluble HLA-DRα2 or by an agonist anti-TIRC7 antibody, initiates an intracellular signaling cascade, which inhibits tbet and Stat4, thereby decreasing Th1 and Th17 proinflammatory cytokine expression, and at the same time increases levels of immune suppressive molecules, including IL-10, CTLA-4, and caspases 9 and 7 (11, 14). Activation of TIRC7 signaling induces antigen specific anergy and inhibits proliferative response in T cells (15).

TIRC7 appears to play a key role in graft versus host disease (16), allograft rejection (17) and autoimmune disorders (9). During solid organ transplant rejection, TIRC7 expression is increased in the allograft and decreased in peripheral blood lymphocytes (18). This dynamic is consistent with highly specialized expression in areas of ongoing inflammation and immune activation. TIRC7 agonist antibodies are effective in prevention and treatment of autoimmune disease and solid organ transplant rejection in preclinical animal models (17). Murine TIRC7+ CD4+CD25lo IL-10 secreting cells were described by Wakkach et al. (19) Although they exhibited immune suppression *in vitro* and *in vivo*, the phenotype of these murine TIRC7+ cells differ from human IR1 cells. Human TIRC7+ T regulatory cells and their characteristics have not previously been elucidated.

Here, we present the phenotype and function of a T-regulatory cell subset, designated IR1 cells, a novel, homogenous, suppressive TIRC7+ T regulatory cell subset. IR1 cells express multiple surface markers associated with immune suppression and cell migration. We have developed agonist antibodies to TIRC7 to expand IR1 cells *ex vivo*, an essential step towards exploring clinical applications.

## Results

### IR1 Cells Are Found in the CD25hi/med Compartment of Tregs

We evaluated the frequency of IR1 cells in peripheral blood from 22 healthy donors. FACS analysis was used to quantify Tregs from peripheral blood mononuclear cells (PBMC). Tregs were identified as CD4+FOXP3+CD25+CD127lo cells. IR1 cells are defined as Tregs that express CD4+FOXP3+CD25+CD127loTIRC7+. The remaining Tregs, “IR1 negative (IR1neg) Tregs”, which do not express TIRC7, are a large heterogeneous population of Tregs (**Supplementary Figure 1.1**).

**Figure 1:**
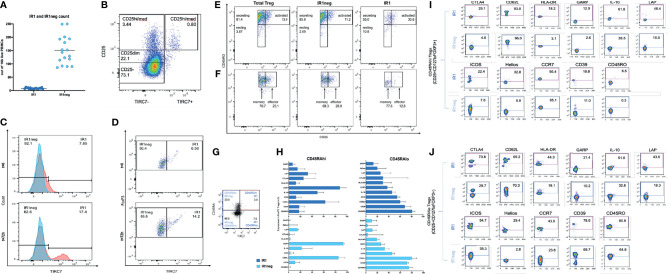
**(A)** IR1 (TIRC7+) and IR1 neg Tregs (TIRC7-) frequencies as counts per 10,000 freshly isolated PBMC in peripheral blood from 22 healthy subjects. **(B)** Differentiation of CD25+ population shows distribution of CD4+ CD25+ TIRC7+ cells, which are mainly localized between CD25med/hi. Cells were pre-gated on CD4. **(C)** Two overlaid histograms (blue FMO control, red TIRC7 panel) show extracellular TIRC7 expression on Tregs from healthy donors, directly after isolation (T=0h) (upper panel) and after 72h IL-2 stimulation (lower panel). The x-axis shows TIRC7 expression. (Supplemental data for gating: 1.1 for t0, 1.2 for t72h). Post expansion (lower panel) the proportion of IR1cells is higher than pre-expansion (upper panel). **(D)** The x-axis shows TIRC7 expression. The Y-axis shows FOXP3 expression levels. At t0 (upper panel) IR1 cells express FOXP3. After 72 hours (lower panel) of IL-2 induction the proportion of FOXP3+IR1 cells is further increased. Cells were pre-gated on CD4, CD25, CD127lo and FOXP3. (**Supplementary Figures 1.2**, **1.3**). **(E)** Treg subpopulations (IR1neg and IR1) are mapped as secreting (CD45RO+, CD25dim/med), resting (CD45RO-, CD25dim/med) and activated (CD45RO+, CD25bright) subsets of Treg at t=72h IL-2 stimulation. (See **Supplementary Figure 1.4** for gating). **(F)** As above, Tregs are mapped to memory (CD45RO+, CD25med) and effector (CD45RO+, CD25hi) subsets of Treg. Cells were pre-gated on CD4, CD25med/high, FoxP3 (**Supplementary Figure 1.1**) The distribution from IR1 and IR1neg to the effector (CD25high, CD45RO+) or memory group (CD25med, CD45RO+) was assessed. **(G)** Expression of TIRC7 vs CD45RA in CD25+CD127loFoxp3+ nonactivated Treg cells. Shown is one representative example out of five obtained from PBMC of healthy donors. IR1 cells make up 11.3% (3.0/26.5) of CD45RAhi, and 9.5% (7.0/73.5) of the more numerous CD45RAlo sets. **(H)** Immune phenotyping of IR1 and IR1neg cells in nonactivated CD45RAhi and CD45RAlo population in HD (n=5) (Gating **Supplementary Figure 1.4**). **(I)** Representative example of activation markers of IR1 and IR1neg Tregs in nonactivated CD45RAhi. **(J)** Representative example of activation markers of IR1 and IR1neg Tregs in nonactivated CD45ROlow in CD25+CD127loFoxp3+ Treg cells. Sample shown obtained from a healthy donors PBMC (n=5). Marker expression is measured on the y-axis versus side scatter (SSC-A) on the x-axis.

IR1 cells represent a small proportion of all PBMC (**Figure 1A**). Tregs represent a mean of 7% of total CD4 cells. IR1 cells in turn, constituted a mean of 5.4% (range 3.3-11) of all Tregs. Circulating IR1 cells represent only about 0.4% of all CD4 cells, with a relatively small inter-individual variability in frequency among the healthy subjects studied here (range 0.2-0.7%) (**Table 1**). IR1 cells had a mean frequency of 8 per 10,000 PBMC (range 5-13) while the mean frequency of IR1neg Tregs is much higher (150 per 10,000 PBMC, (range 86-250). Per 10,000 PBMC, the mean within-subject ratio of IR1neg Tregs to IR1 cells was 21 (range 8 to 47). Within healthy subjects, the frequency of IR1 cells was not correlated with the frequency of IR1neg Tregs per 10,000 PBMC. TIRC7+ Tregs (IR1 cells) are shown mapped to the CD25 hi-med compartment of total Treg cells (**Figure 1B**).

**Table 1:** Relative cell frequencies. Healthy Donors (n=17).

	Cell/Parent	
CD4/PBMC %	Mean (SD)	22.65 (7.98)
	Median	23.60
	Min- Max	9.52- 34.80
Tregs/CD4%	Mean (SD)	7.39 (2.2)
	Median	7.18
	Min- Max	4.70-11.90
IR1/Tregs %	Mean (SD)	5.35 (2.26)
	Median	5.56
	Min- Max	3.35-10.6
IR1/CD4%	Mean (SD)	0.4 (0.15)
	Median	0.38
	Min- Max	0.21-0.73
IR1/10,000 PBMC	Mean (SD)	8.01(2.56)
	Median	7.50
	Min- Max	5.04-12.97
IR1neg Treg/10,000 PBMC	Mean (SD)	150 (53)
	Median	143
	Min- Max	86-250

### IL-2 Disproportionally Expands IR1 Cells

Treg cells were activated *ex vivo* by incubation with IL-2 for 72 hours for 8 of the subjects. IL-2 incubation disproportionally increased IR1 cells. Two overlaid histograms (blue FMO control, red TIRC7 panel, **Figure 1C**) show the TIRC7 expression of Tregs from a healthy donor directly after isolation (t0) and after IL-2 activation (t72h). This corresponds to the FOXP3+ population of resting and activated Tregs (**Figure 1D**). Activation of Tregs with IL-2 for 72h resulted in a substantial increase in the proportion of IR1cells (17%) in comparison to baseline (8%).

The IR1 cell phenotype was found to be distinct and stable. After activation, IR1 cells continued to demonstrate stable, high expression of FOXP3. In the example shown (**Figure 1D** and **Supplementary Figures 1.2**, **1.3**), at t=0h, among CD25hiFOXP3+cells, 6.56% were TIRC7+. After *in vitro* expansion with IL-2 (1,000 UI/ml) for 72h, IR1 cells made up 14.2% of CD25hiFOXP3+cells.

At baseline (t=0), mean IR1 cell count for these subjects was 8.5 per 10,000 per PBMC and by t=72 hours it had increased to 30 per 10,000 (**Table 2**). By contrast, the mean for IR1neg Tregs at t=0 was 124 per 10,000 PBMC and at 72 hours had increased to 152 per 10,000 PBMC. While IR1 cells increased by a mean of 3.5-fold (range 2 to 7), IR1neg Tregs increased by a mean of 1.3-fold (range 0.8 to 2). The average within-subject expansion was 3 times higher for IR1 cells than IR1neg Tregs. The ratio of IR1neg Tregs to IR1 cells, was 15 at baseline and declined to 5 at 72 hours.

**Table 2:** Mean IR1 cell and IR1neg Treg counts per 10,000 live PBMC after isolation (t=0h) and after activation with IL-2 (t=72h). N=8 subjects.

Time point	IR1 cells	IR1 neg Tregs	IR1neg/IR1 ratio
**t=0h**	8.49	123.8	14.6
**t=72h**	30.01	152.2	5.1
Mean-fold increase	3.53	1.23	

IR1 Tregs can be divided into activated/secreting/resting subpopulations based on surface markers. The proportion of IR1 Tregs in the secreting (CD4+CD25dim/medFoxP3CD45RO+), activated (CD4+CD25hiFoxP3CD45RO+) and resting (CD25dim/med, CD45RO-) populations are 55%, 30.6% and 10.8%, respectively. For IR1neg Treg the proportions are 85.8% secreting, 11.2% activated and 2.7% resting, respectively. Thus, IR1 Tregs are disproportionately found in the activated population of Treg cells (**Figure 1E**). As shown in **Figure 1F**, IR1 Tregs locate less often (12.8%) to effector Treg compartments compared to IR1neg Treg cells (28.0%). (Supplemental data for gating 1.1 for t0, 1.2 for t72h).

IR1 cell frequency and marker expression were analyzed in non-activated CD45RAhi and CD45RAlo Treg subsets (**Figures 1G**–**J** and **Supplementary Figure 1.4**).

IR1 cells are about 11.3% (3.0/26.5) of CD45RAhi, and 9.5% (7.0/73.5) of the more numerous CD45RAlo cell subset (**Figure 1G**).

The surface markers expressed in IR1 cells were generally higher on CD45RAlo IR1 cells (**Figures 1I, J**).

### IR1 Cells Combine Homing and Anti-Inflammatory Markers on Their Surface

**Figure 2:**
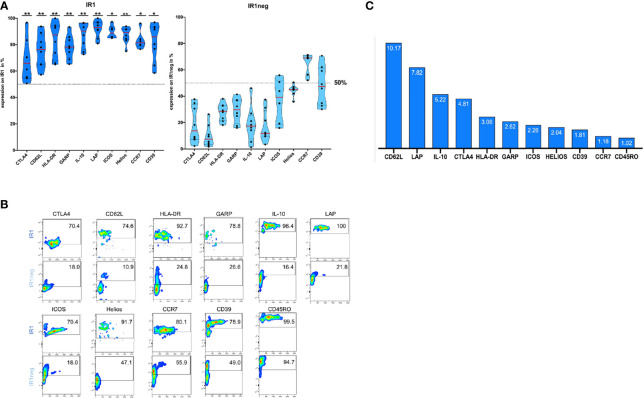
**(A)** IR1 cells express multiple membrane and soluble immune suppressor molecules. Surface expression of HLA-DR, CD39, LAP, ICOS, IL-10, CTLA-4, CCR7, CD62L, GARP and Helios is higher for IR1 cells than for IR1neg Tregs after 72h IL-2 induction. Violin plots were generated using statistical software Prism GraphPad. (Gating **Supplementary Figure 1.2**, for overlaid histograms from marker on IR1 versus IR1neg; **Supplementary Figure 2A**). Medians indicated by red lines. P-values reflect **P < 0.01, *P < 0.05 whether the ratio of IR1/IR1neg is significantly different from one. (See also **Table 3**). **(B)** Representative example of Immunophenotyping for two subpopulations: IR1 Treg compared to IR1neg Treg, using the above ten markers plus CD45RO. Marker expression is measured on the y-axis versus side scatter (SSC-A) on the x-axis. Smoothed pseudo-color dot plots are shown. (Gating **Supplementary Figure 1.2**, for overlaid histograms from marker on IR1 versus IR1neg; **Supplementary Figure 2A**, MFI- Values **(B)**. **(C)** Ratio (IR1 cells/IR1neg Tregs) of median percentage Tregs with the respective marker: CD62L, LAP, IL-10, CTLA-4, HLA-DR, GARP, ICOS, HELIOS, CD39, CCR7 and CD45RO.

After 72h of IL-2 stimulation, Tregs were stained with antibodies against surface molecules and phenotyped using FACS analysis. IR1 cells proved to be phenotypically distinct from IR1neg Tregs with respect to multiple markers associated with immune suppressive activity. **Table 3** shows percent surface expression (median and inter-quartile ranges) of key surface markers for IR1 cells and IR1neg Tregs after 72h of IL-2 *ex-vivo* exposure. Highly significant differences were noted for CD62L, LAP, IL-10, CTLA-4, HLA-DR, GARP, Helios (p<0.01) and for ICOS, CD39, CCR7(p<0.05). Violin plots of these data (**Figure 2A**) make clear the distinct overall patterns of surface expression and the differences in individual markers frequency for IR1 cells versus IR1neg Tregs. Representative flow cytometry examples are shown in **Figure 2B**.

**Table 3:** Median and inter-quartile ranges (IQR) of percent marker expression on IR1 and IR1neg Tregs after 72h *in vitro* IL-2 exposure of PBMC.

HD (n=8)	IR1 cells			IR1neg Tregs			IR1/IR1neg cell ratio	
	1. Quartile	Median	3. Quartile	1. Quartile	Median	3.Quartile	Ratio	p<0.01**, p<0.05*
**CD62L**	65.1	**76.3**	83.8	3.9	**7.5**	12.7	10.17	0.0078 **
**LAP**	89.4	**93.0**	96.8	9.8	**11.9**	24.0	7.82	0.0078 **
**IL-10**	79.4	**90.3**	96.3	12	**17.3**	22.6	5.22	0.0078 **
**CTLA-4**	55.3	**65.9**	73.7	7.9	**13.7**	31.6	4.81	0.0078 **
**HLA-DR**	71.9	**87.5**	93.4	22.7	**28.6**	32.8	3.06	0.0078 **
**GARP**	73	**78.1**	84.0	23.1	**29.8**	36.8	2.62	0.0078 **
**ICOS**	85.8	**88.8**	92.3	23.1	**39.3**	49.1	2.26	0.0312 *
**HELIOS**	84	**91.5**	91.7	43.4	**44.9**	46.2	2.04	0.0078 **
**CD39**	73.7	**86.0**	93.2	34.2	**47.5**	59.9	1.81	0.0156 *
**CCR7**	79.7	**81.4**	83.8	59.4	**69.0**	70.2	1.18	0.0312 *
**CD45RO**	97.9	**98.3**	99.0	84.7	**96.5**	99.0	1.02	0.0859 ns

Wilcoxon matched pairs, sign-rank test was used to compare median percent positive IR1 and IR1neg Tregs for each marker. IR1/IR1neg Tregs ratio of percent cells positive for the marker.

*p < 0.05.

**p < 0.01.

ns, not significant.

While the above-mentioned markers were more frequently expressed on IR1 cells than IR1neg Tregs, the magnitude of the difference varied by marker, largely due to variable expression on IR1neg Tregs. **Figure 2C** shows the ratio of positive IR1 cells to IR1neg Tregs for each marker. Highest is CD62L, with a ratio of 10. CD62L is expressed by a median 76% (IQR 65 to 84) of IR1 cells, but on only 7.5% (IQR 4 to 13) of other Tregs (**Table 3**).

**Figure 3:**
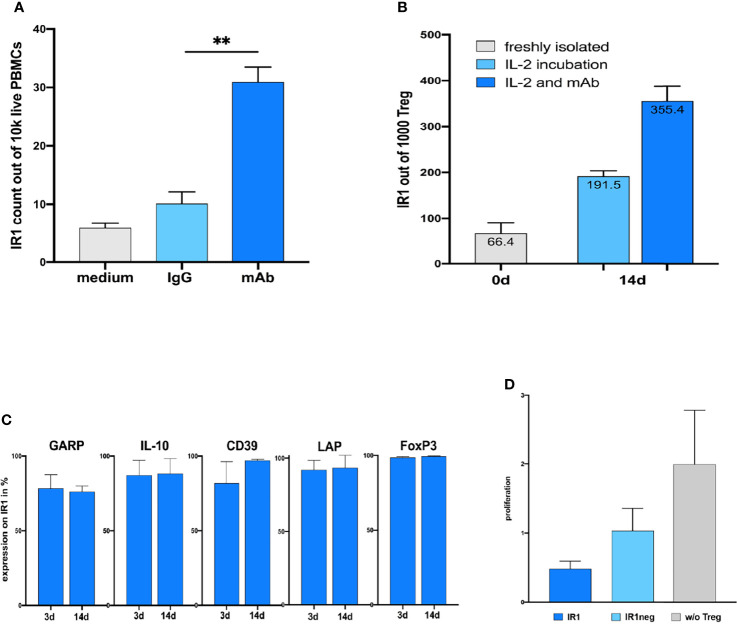
**(A)** Coincubation of humanized TIRC7 agonist antibody significantly induce TIRC7+ IR1 cells in human PBMC cultures after 72h compared to IgG-control *ex vivo* (n=7). ** mAb = p.0025. **(B)** Exposure of purified CD25+CD4+ cells to IL-2 plus agonist anti-TIRC7 mAb leads to profound expansion of IR1 cells after 2 weeks. The absolute numbers of purified Treg cells after 2 weeks of expansion cultures are shown. The absolute numbers of IR1 cells in the presence of TIRC7 antibody plus IL-2 exceed those achieved in the IL-2 cultures alone by several fold. **(C)** Stability of suppressive phenotypes as reflected based on the expression of markers LAP, CD39, GARP, IL-10 and FOXP3 remain high and unchanged in expanded IR1+ cultures after 14 days. **(D)** In BrdU proliferation assay, expanded human IR1+ cells show more potent inhibition of lymphocyte activation in mixed lymphocyte culture (MLC) compared to IR1neg Tregs.

Key suppressive markers are present on a high proportion of IR1cells. LAP is expressed by a median of 93% of IR1 cells compared to 12% of IR1neg Tregs. Similarly, IL-10 is found on 90% of IR1 cells and 17% of IR1neg Tregs (**Table 3**). The constellation and concentration of known cell immune suppression markers on IR1 cells is strikingly and significantly different from IR1 neg Treg cells. These results were confirmed in purified and expanded Treg cultures as shown in **Figure 3**.

### Agonist TIRC7 Antibody Disproportionally Expands IR1 Cells Beyond Levels Achieved With IL-2, While Retaining the Suppressive Phenotype

Prior work has shown that anti-TIRC7 agonist antibodies stimulate human CTLA4+ Tregs, while inhibiting Th1 and Th17 T-effector cells (20). An anti-TIRC7 IgG1 agonist antibody, humanized *via* the standard CDR grafting method (21) was selected based on binding affinity and specificity for TIRC7 (**Supplementary Figure 3**). It demonstrated specific binding to TIRC7 expressed in COS7 cells (**Supplementary Figure 3A**) or to wild type mice (+/+) but not to TIRC7 deficient mice splenocytes (-/-) and (**Supplementary Figure 3B**, left panel) with an affinity at 200 – 300 pM.

TIRC7 antibody was used to expand and isolate IR1 cells from purified Tregs obtained from 3 healthy subjects and to characterize the number of TIRC7+Tregs (IR1 cells) in the culture *via* flow cytometry. Incubation with this antibody plus IL-2 induced TIRC7+ IR1 Tregs to over 30%, substantially exceeding induction *via* IL-2 only (**Figure 3A**). Stability of the IR1 Treg phenotype was examined for these subjects after 14 days expansion (**Figure 3B**). High expression levels of LAP, CD39, GARP, FOXP3 and IL-10 were also maintained after two weeks expansion in IR1 cells (**Figure 3C**).

To evaluate the overall immunosuppressive activity of the 14-days expanded and purified IR1 cells, we assessed their ability to inhibit cell proliferation in a mixed lymphocyte reaction (MLR) as compared to IR1neg Tregs. Human T-effector cells (Teff) and allogeneic dendritic cells (DC) were stimulated in an MLR under three conditions: (1) Teff + DC + IR1 cells; (2) Teff + DC +IR1neg Tregs; (3) Teff + DC (control). IR1 cells reduced proliferation by 80% compared to the control without Tregs, while IR1neg Tregs showed only a 50 percent reduction. Thus, IR1 cells show greater functional suppression compared to IR1neg Tregs (**Figure 3D**, from left to right).

## Discussion

Tregs consist of a heterogeneous pool of cells and play an important role in limiting inflammatory processes. T-regulatory suppressive activity is one of the most powerful tools for modulating the immune system and is a recognized target for clinical intervention.

Here we highlight for the first time, the critical potential of IR1 cells as a distinct subset of Treg cells, defined by surface expression of the immune modulatory protein TIRC7. IR1 cells, make up some 5- 10% of T-regulatory cells in healthy individuals and exhibit multiple phenotypic markers with distinct mechanisms of immune suppressive action. Flow cytometry panels used CD4+FOXP3+CD25+CD127lo staining by incorporating anti-TIRC7 antibodies to identify Tregs with surface expression of TIRC7.

Within non-activated Tregs, IR1 cells mapped to CD45RAhi and CD45RAlo subset, however with an increase in the CD45RAlo fraction. Expression analysis of TIRC7 (alias TCIRG1) as shared in gene expression databases, for human immune cell populations (Database of Immune Cell Expression, Expression Quantitative Trait Loci (eQTLs) and Epigenomics, www.dice-database.org) as well as Immgen (www.immgen.org) support our classification of TIRC7+Tregs to the resting and activated subsets of Tregs. The databases contain resting/naïve Treg populations and an activated/memory Treg population for gene expression analysis. They use the same gating strategy for isolation (resting/naïve: CD4+, CD25 hi, CD127 lo, CD45RA+; activated/memory: CD4+, CD25 hi, CD127 lo, CD45RA-as used in our experiments. Our findings are in agreement with these databases which also show higher expression of TIRC7 in activated/memory Tregs compared to resting/naïve Tregs (DICE: mean of 185.59 to 71.62 in TPM, Immgen: normalized expression value of 373.143 to 217.560) isolated from human PBMC.

Flow cytometric analysis of the IR1+ subset, shows that they fall within the FOXP3hi compartment. FOXP3, the master regulator transcription factor, is responsible for restricting immune self-reactivity and suppressing excessive inflammation. FOXP3 acts by repression of gene transcription for IL-2 and other inflammatory cytokines, while activating suppressive pathways *via* transcription of IL-2RA, CTLA4, and the FOXP3 gene itself (22, 23). FOXP3 expression is a determinant of immune homeostasis and is directly relevant to the stability of the cell lineage. Ablation of a conditional FOXP3 allele in mature Treg cells results in generation of “former” Treg cells that may acquire proinflammatory properties. Tregs are an actively dividing cell population with a critical role in containing immune-mediated inflammation. Ongoing Treg self – renewal, combined with stable FOXP3 expression is essential to maintaining immune homeostasis (24)

Tregs in peripheral blood express CD45RO, suggesting that these cells were previously activated and induced to proliferate. CD45RO cells are known to be extremely proliferative, with 20% in cycle at any given time compared to 2% of CD45RA naïve (resting) Tregs (25). IR1 cells map to the activated Treg subset, that manifests the highest proliferation and most suppressive properties (5). These finding are consistent with the previously described immune suppressive role of TIRC7 after T cell receptor activation.

In agreement with this notion, IR1 cells demonstrate superior inhibition of cellular proliferation *in vitro*. IR1neg Tregs, while much more numerous, are phenotypically heterogeneous and demonstrate less suppressive activity than IR1 cells.

*Ex vivo* incubation of lymphocytes with IL-2 yielded a 3.5-fold increase of IR1 cells, compared to a 1.3-fold increase in IR1neg Tregs at 72 h. The addition of TIRC7 agonist antibody led to a marked further shift towards IR1 cells.

The expanded IR1 cells continue to display the same high level of suppressive surface markers at 14 days as observed after 3-day activated cultures. IR1 cells expanded in the presence of the agonist TIRC7 antibody, maintain the potent suppressive phenotype reflected by the surface markers. It is plausible that differential expansion of IR1 cells also occurs *in vivo* (26). While low dose IL2 treatment expands the heterogenous Treg population, anti-TIRC7 antibody is likely to be highly selective in expanding IR1 cells. The importance of selective expansion of IR1 cells, is supported by the observation that the success of therapeutic models of heart transplant using agonist TIRC7 antibodies is associated with induction of suppressor pathways and inhibition of effector cells (27).

The IR1 cell phenotype is distinctive, with key surface markers occurring on a high percentage of IR1 cells, ranging from a median of 65.9% for CTLA4 to 93.0% for LAP. By contrast, for IR1neg Tregs, these markers range from a median of 7.5% for CD62L to 69.0% for CCR7 (**Table 3**). Only CD45RO, does not differ, being present on a median of 98.3% of IR1 and 96.5% of IR1neg Tregs.

While each suppressive marker is significantly more frequent on IR1 cells compared to IR1neg Tregs, the degree of difference, expressed as the ratio of percent frequency, ranges from a high for CD62L of 10.2 to a low for CCR7 of 1.2. The potential impact of individual markers and combinations of markers is considered below.

CD62L and CCR7 both play a role in Treg homing to lymph nodes and sites of inflammation (28, 29). CD62L is an important T cell homing receptor which orchestrates T cell trafficking of Tregs to secondary lymphoid organs. CCR7’s multifaceted effects include its impact on the migration of lymphocytes to areas of inflammation (30, 31). IR1 cells express both these markers: a median of 76.3% for CD62L and 81.4% for CCR7. However, activated IR1neg Tregs do not frequently express CD62L (median 7.5%) resulting in a high ratio (10.2) for IR1 to IR1neg Tregs. For CCR7 the picture is different since a median of 69.0% of IR1neg Tregs express CCR7. However, the combination of CD62L and CCR7 will be much greater for IR1 cells and would potentially increase homing characteristics (32).

LAP+GARP+FOXP3+ Tregs are particularly effective in suppressing the proinflammatory effects of conventional T cells. Human LAP+GARP+FOXP3+ regulatory T cells attenuate xenogeneic graft versus host disease (33, 34). Majority of IR1 cells express these markers, a median of 93.0% for LAP and for 78.1% for GARP. By contrast, on IR1neg Tregs, LAP is expressed by a median of 11.9% and GARP by a median of 28.9%. Thus, the combination of LAP and GARP should occur much more frequently on IR1 cells than IR1neg Tregs. The combination of LAP and GARP is thought to increase suppression of conventional T-cells.^25^

IL-10 is specifically related to the inhibition of proliferation in MLC. It plays an important role in the suppressive function of antigen specific, induced Tregs through its effects on dendritic cells (23, 35). A median of 90.3% of IR1 cells express IL-10 compared to 17.3% of IR1neg Tregs. Mixed lymphocyte cultures with IR1 cells secreted high levels of IL-10. Such secretion was not seen for IR1neg Tregs.

CTLA-4 inhibits dendritic cell maturation, a core mechanism of T regulatory cell mediated suppression. The co-stimulatory and co-inhibitory molecules CTLA-4 and ICOS are involved in the pathogenesis of autoimmune disease (36, 37). TIRC7 is involved in regulation of CTLA-4, and its activation leads to increased CTLA-4 expression (11, 38, 39). IR1 cells show higher expression of both CTLA-4 and ICOS than do IR1neg Tregs. CTLA-4 is expressed by a median of 65.9% (of IR1 cells compared to 13.7% of IR1neg Tregs. The ratio of median percent cell expression for CTLA-4 is 4.81. ICOS is expressed by 88.8% of IR1 cells compared to 39.3% of IR1neg Tregs with a resultant ratio of nearly 2.

HLA-DR+ Teff cells expressed significantly higher levels of Th1/Th17 cytokines that may regulate Treg function through a reciprocal counter-balancing relationship. Activated HLA-DR+CD4+ T cells may contribute to inflammation by compromising Treg-mediated suppression. IR1 cells provide potent inhibition of Teff activation *via* enriched expression binding to HLA-DR + Teff cells. Most IR1 cells express HLA-DR: a median of 87.5%. By contrast a median of 28.6% IR1neg Tregs are HLA-DR+, resulting in a ratio of 3.6.

Two major subsets of Treg cells have been described *in vivo*; thymus derived Treg (tTreg) cells, which develop in the thymus, and peripherally induced Tregs (pTreg). Helios, a member of the Ikaros transcription factor family, is expressed in 60-70% of Tregs and is believed to be a marker of thymus derived Tregs which are stable and highly suppressive. The presence of Helios influences Treg stability and function (26). For IR1 cells the median Helios expression is 91.5% compared to 44.9% for IR1neg Tregs, a ratio of 2.04.

CD39, an ectonucleotidase, which hydrolyses pro-inflammatory ATP, is regarded as a marker of highly active and suppressive T regulatory cells. Recent studies have suggested that catalytic inactivation and conversion of the extracellular ATP and ADP to AMP, both induced by CD39, serve as anti-inflammatory mechanisms by which Tregs mediate immune suppression in human autoimmune diseases. Median expression of CD39 on IR1 cells was 86.0%, significantly higher than 47.5% on IR1neg cells, with a resulting ratio of 1.81.

*In vitro* suppression assays reflect cell suppressive activity *in vivo*, with networks of pathways working separately but in parallel, to achieve suppression. A classic example is Treg cell-mediated suppression in mixed lymphocyte reactions. In this manuscript we have shown that IR1 cells reduce lymphocyte proliferation in MLR by over 80%, versus less than 50% for IR1neg Tregs (**Figure 3B**). The frequency of IR1 cells in peripheral blood is narrowly maintained at a low level, with a mean of 8 per 10,000 PBMCs. IR1 home to sites of inflammation, and the *in vivo* expansion of IR1 cells, directly or indirectly will multiply this effect.

As shown in **Figure 3A**, co-culture of CD25+ CD4+ T cells with the agonist TIRC7 antibody for 14 days, triggered additional induction of IR1 cells beyond IL-2 alone. These TIRC7+ cells showed high, stable expression of LAP, CD39, IL-10, GARP and FOXP3, underlining the antibody’s ability to expand and still maintain the phenotype. The agonist antibody not only expands IR1 cells, but the expanded cells over weeks maintain a stable and suppressive phenotype.

However, CD4+CD25+CD127loFOXP3+ Tregs, are heterogeneous, with different phenotypic and functional characteristics. Although polyclonal Treg treatments have been promising in terms of safety, the efficacy of such Treg therapy has been inconsistent. This may reflect the heterogeneity of the Treg cells, not all of which are stable or immune suppressive. Ultra-low dose IL-2 has been used *in vivo* to selectively expand Treg expressing high affinity IL-2 receptors. This approach has been well tolerated in various disorders (40).

Studies in diverse indications suggest a therapeutic potential for Tregs in autoimmune disease and solid organ transplantation (TRACT). To work effectively, adoptively transferred Tregs must migrate to and survive within the inflamed tissue. The safety and liver-homing properties of GMP grade autologous Tregs have been demonstrated in patients with autoimmune hepatitis (41) and shown Tregs migrated to liver suggesting autoimmune liver diseases. Based on their phenotypic markers, IR1 cells are expected to be found at sites of inflammation. In fact, data from an ongoing study displayed IR1 cells in human autoimmune samples (unpublished data, manuscript in preparation). TIRC7 positive cells are rare in peripheral blood and concentrated in mesenteric lymph nodes and small intestinal sub-mucosa as shown by Wakkach et al. (19) Despite the different phenotype between mice and human IR1 cells it has been shown that murine IR1 type cells express high amounts of IL-10 and exhibit a highly inhibitory Tr1 phenotype (19).

The IR1 cell subset of Tregs is stable, relatively homogeneous and demonstrates a highly suppressive phenotype. These cells can be readily induced, expanded and isolated and have therapeutic potential in a wide range of indications. In healthy subjects the ratio of IR1 cells to PBMC lies within a narrow range. IR1 cells can use multiple immune suppressive tools such as cell-cell contact inhibition *via* CTLA4, direct cytotoxicity *via* CD39 and inhibition of proliferation *via* soluble mediators such as IL-10. High levels of CD62L and CCR7 can direct IR1 cells to lymphatic tissue and areas of inflammation. IR1 cells may play the role of a readily mobilizable population in the peripheral blood that can be dispatched to areas of excessive immune activation. IR1 cells in the peripheral circulation constitute a rapidly inducible “ready-to-go” population. Expanded IR1 cells remain stable and express similar levels of activation markers on their surface for some weeks.

IR1 cells are found among non-activated CD45RAhi cells as well as CD45RAlo Tregs, an observation that supports the presence of a specific lineage defined by TIRC7 expression. Non-activated CD45RAlo IR1 cells show marked expression of suppressive molecules. However, the differential expression of the suppression markers between IR1 and IR1neg Tregs is most pronounced after activation. After IL-2 activation, respective markers are expressed on 66-93% of IR1 cells. For the IR1neg population, expression is dramatically lower for each molecule measured (**Figure 2A**). It is plausible that circulating IR1 cells, armed with a cargo of suppressive markers, are deployed to sites of immune activation and play a key role in maintaining immune homeostasis in inflamed tissue. By contrast, the potential impact of highly suppressive cells is limited physiologically by maintaining low numbers of IR1 cells in the peripheral circulation. IR1 cells have the potential to restore immune homeostasis in diseases that are characterized by inadequate T regulatory cell activity. This approach has the potential to modify disease natural history without requiring ongoing immune suppressive treatment. Amplification of autologous IR1 cell activity, can suppress excess T effector cell activity directed at a specific target. Autoimmune diseases (e.g., Type 1 diabetes, autoimmune liver diseases) as well solid organ transplant are potential indications for IR1 cell therapy.

A high level of immune suppression is achieved by IR1 cells, possibly through its ability to use multiple mechanisms, such as inhibition of activation *via* cell-cell contact, soluble mediators, or direct induction of apoptosis. Thus, both, antibody targeting of TIRC7 or cell therapy using IR1 cells, offer disease depended distinct therapeutic options.

While the healthy subject cohort studied is not large, the characteristics of IR1 cells are quite consistent. Subjects were identified by a “convenience sample” that was not diverse or representative, particularly with respect to age. It is not yet known whether IR1 cells from pediatric or geriatric populations will show similar features. IR1 cells studied were derived from peripheral blood. Such TIRC7+ Tregs can be seen in histopathological samples from various autoimmune diseases (data on file). In addition, characteristics of IR1 cells in subjects with acute or chronic diseases are currently under study with the goal of unlocking the therapeutic potential of IR1 cells.

## Methods

### Subjects

22 healthy Caucasian donors participated, 12 female and 10 males, mean age 24.9 years.

### Ethics Statement

Collection of blood from healthy volunteers and tissues from patients was performed after approval by the local ethical committee and signed informed consent (Charité Universitätsmedizin, reference number EA2/095/18).

### Peripheral Blood Mononuclear Cell Isolation

Peripheral blood was collected in four 10ml BD vacutainer EDTA tubes **f**rom each enrolled subject and processed immediately. PBMC were isolated using Ficoll density gradient centrifugation. The blood was transferred into two 50ml Falcon tubes and diluted with 15ml PBS per tube to increase purity. The diluted blood was then carefully layered on top of 15ml Ficoll Plaque solution and centrifuged at 4°C, for 20 minutes at 300g without a break. Due to the different densities of the components, the suspension separates, and the leukocytes form a distinct layer between the plasma and Ficoll layer. After pipetting 10ml of the overlaying plasma, PBMCs were isolated using a 7ml plastic transfer pipette. ACK-lysis buffer was then added, and the suspension was centrifuged. Cells were counted with a Neubauer counting chamber and seeded in a 96-well plate.

### IL-2 Stimulation

2x10^5^ PBMCs per well were cultured for 72 hours in 200µl RPMI medium with 10% FCS, 1% Penicillin/Streptomycin (P/S), 1% L-Glutamine and 1,000 IU/ml recombinant IL-2, in a 96 well flat bottom plate at 37°C and 5% CO2.

### Humanized Antibody Generation

TIRC7 mAb was humanized *via* CDR grafting (42) and characterized for specificity, affinity (unpublished, Nekonal Sarl, data on file) and purified antibody 10 µl/ml was used for expansion cultures.

### Treg Isolation

The experiments were performed using the Human CD4+ CD25+ Regulatory T Cell Isolation Kit, from Miltenyi. Freshly isolated PBMCs were centrifuged for 10 minutes at 300g. The supernatant was discarded, and the cell pellet resuspended in 90µl MACS buffer. 10µl of CD4+ T Cell Biotin- Antibody cocktail was added, and cells incubated for 5 minutes in the refrigerator. 20µl Anti-Biotin Microbeads were applied and incubated for 10 minutes. To isolate CD4+ from CD4- cells, magnetic depletion separation using an LD column was performed. CD4+ cells were then collected in the effluent, while CD4- cells remained in the column. The effluent was collected and centrifuged for another 10 minutes at 300g. After aspirating the supernatant, the cell pellet was resuspended in 90µl buffer, and 10µl CD25 microBeads were added and incubated for 15 minutes in the dark, in the refrigerator. Cells were then magnetically separated and positively selected by using an MS-column.

### Treg Expansion

Expansion of Tregs was performed by using the Human Treg Expansion Kit, from Miltenyi. Isolated CD4+ CD25+cells were washed twice with MACS buffer and then counted in a Neubauer counting chamber. 1x10^5^ Treg per well were seeded in a 96-well flat bottom plate with 200µl of TexMACS-Medium with 10% human AB serum, 500 IU/ml IL-2 and 100 ng/ml Rapamycin. To stimulate the cells, 20µl CD3/CD28 MACSiBead Particles were added to each well. Depending on growth and medium consumption, cells were split and transferred into a 48 flat bottom well plate with fresh medium.

### IR1/IR1neg Treg Cell Isolation

Expanded Treg were collected, washed with MACS buffer, and purified by removing the MACSi beads using a MACSiMAG Separator. To isolate IR1 (TIRC7+ Treg) and IR1neg (TIRC7- Treg), the cells were stained with 2µl Biotin labeled anti-TIRC-7 antibody (conc.20µg/ml) for 30 minutes, at 4°C i in the dark. After adding 20µl anti-Biotin Microbeads and incubating for 10 minutes at 4°C in the dark, the cells were divided into TIRC7+ and TIRC7- Treg by magnetic separation through a MS-column.

### Mixed Lymphocyte Reaction

20,000 IR1 or 20,000 IR1neg cells respectively, were cultured for 5 days with 20,000 responder T-cells (CD4+ CD25-) and 4,000 allogenic dendritic cells (DCs). Isolation was performed, using Miltenyi’s DC isolation Kit, in 200µl MLR-Assay Medium (TexMACS medium with 10% human AB serum) in a 96 well flat bottom plate.

### Proliferation Assay

After 5 days, per well, 20 µl of the BrdU labelling reagent diluted 1:100 in MLR-assay medium were added to each well to reach a final BrdU dilution of 1:1,000. The cells were incubated for another 16h at 37°C and 5% CO2. After the second incubation, the 96 well plate was centrifuged (300 g, 4 minutes, 4°C) and the supernatant discarded. The proliferation assay was performed using a commercial kit by Roche (11647229001) according to the manufacturers protocol. Finally, 10 µl stop solution was added per well and the optical density at 450 nm was measured using the SUNRISE™ Microplate Reader by Tecan.

### Flow Cytometry

Analysis was performed at two different timepoints to examine distinct activation levels of Treg (t0 – resting Treg, t72h – activated Treg). 2x10^5^ freshly isolated PBMCs per well were Fc-blocked (5µl/well), stained with TIRC7 mAb3 (FITC, anti-mouse, 20µg/ml) for 1h at room temperature (RT) in the dark and then incubated with the extracellular antibodies CD3 (BV510), CD4 (AF700), CD25 (APC), CD127 (BV605) from BioLegend in a concentration of 1:200 for 20 minutes at RT in the dark. After washing and centrifuging, the cell pellets were resuspended in 200µl MACS buffer plus 7-AAD (1:100) to exclude damaged or dead cells. 2x10^5^ freshly isolated PBMCs per well were seeded in a 96-well flat bottom plate for the 72h incubation and *in-vitro* IL-2 stimulation. At 72h the stimulated PBMCs were transferred to a 96 well round bottom plate to reduce cell loss. Cells were incubated with 5µl Fc block per well for 10 minutes to prevent unspecific binding. After centrifuging with 300g for 4 minutes at 4°C, discarding the supernatant and resuspending the cell pellets in 100µl MACS buffer, the FITC-labelled TIRC7 mAb3 was added and the well plates were incubated in the dark at RT for 1 hour.

Thereafter the cells were centrifuged as above, washed with 200µl MACS buffer per well and then dyed with 1µl fixable live dead marker (Thermo Fisher Scientific or Viobility 405/452 from Miltenyi) in 100µl PBS per well for 30 minutes in the dark at RT. The master mixes (dilution 1:200 in MACS buffer) for subsequent extracellular staining were prepared during the incubation times and included the following markers: CD4 (AF700, APC-Fire), CD25 (APC, PE-Cy7), CD127 (BV605), GARP (APC), CTLA-4 (BV605), LAP (PE), CD45RO (BV711), HLA-DR (PE-Cy7), CD39 (PE-Cy7), CD62L (AF700), CCR7 (APC), ICOS (BV421). After 30 minutes, the well plates were centrifuged (300g, 4 minutes, 4°C), supernatants were discarded, cell pellets resuspended in 100µl per well of the master mixes and incubated for 20 more minutes, as above. Processed PBMCs were washed with 200µl MACS buffer and then fixed and permeabilized following the BioLegend Fix/Perm buffer set protocol to stain the intracellular markers FOXP3 (PE, BV421), IL-10 (PE) and Helios (APC) for 20 minutes in the dark at RT (see Supplemental data for panels and antibodies). After 2 washing steps, the cell pellet was resuspended in 200µl MACS buffer, and the samples were then measured using the BD LSR Fortessa. For cytokine analysis Golgi inhibitor Brefeldin A 1000X was added to the cells in culture at a concentration of 1X (diluted in culture medium) according to manufacturer’s protocol (Biolegend) 6 hours before harvesting and staining for flow cytometric analysis.

Stopping gates were set on live cells, 1x10^5^ live cells after isolation and 2-5x10^4^ after *in vitro* stimulation. To ensure the correct gating for TIRC7, fluorescence minus one (FMO) control without TIRC7 mAb was used for each Treg panel. To compensate for spectral overlaps between different fluorophores, samples were single stained with each antibody. Data reproducibility and instrumental quality control was achieved by acquisition of cytometer Setup &Tracking beads from BD and 8 peak Rainbow Calibration Beads from BioLegend. FACS data was analyzed using FlowJo 10.7.1 Software. Treg and their subsets were identified as reported) (see Supplemental data for gating). Treg count, TIRC7+/- Treg count, marker expression on the respective subsets and MFI (mean fluorescent intensity) were analyzed.

### Immunohistochemistry

Paraffin sections were dewaxed and hydrated prior to epitope retrieval at pH6. After blocking of endogenous peroxidase (Dako REAL Peroxidase Blocking Solution (Agilent, # S202386-2) sections were incubated with anti-FOXP3 (clone PCH101, ThermoFisher Scientific, 1:50) for 30 minutes at room temperature followed by incubation with secondary antibody (rabbit anti-rat (1:1,000, Dianova # 312-005-003) for 30 minutes at RT. For detection, EnVision+ Single Reagent (HRP. Rabbit) (Agilent # K400311-2) was used. Sections were subjected to epitope retrieval at pH8 prior to incubation with anti-TIRC7 (CellAct, 1:500) for 30 minutes at RT. For detection, the LSAB system was used (Dako REAL Detection System, Alkaline Phosphatase/RED, Rabbit/Mouse (Agilent #K500511-2). Nuclei were stained using Mayer´s hemalaun solution (Merck Millipore # 1092490500) and sections cover-slipped with Kaiser´s glycerol gelatin (Carl Roth GmbH # 6474.1).

### Confocal Microscopy

COS7 cells were transfected with 6µg pcDNA-TIRC7-myc and pcDNA3-myc as control. After transfection the expression of recombinant TIRC7 protein was analyzed in transfected COS7 with pcDNA3-TIRC7-myc (COS7^t^) and untransfected (COS 7nt) with anti-TIRC7 mAb17. Immunostaining with anti-myc antibody (myc-Ab) (Oncogene) was used as positive control for the transfection. Cells were incubated overnight at 4°C with anti-TIRC7 mAb (10µg/ml) and anti-myc mAb´s (Oncogene,1/10) rinsed with PBS, and incubated with Cy2-conjugated goat anti-mouse IgG (Dianova,1/250) or goat anti-human IgG (Sigma, 1/100) diluted in 5% milk/PBS. Cells were mounted with 50% (v/v) glycerol/PBS and analyzed by confocal laser scanning microscopy.

Wild types and TIRC7 knockout mice splenocytes were fixed for 20 min at 4°C with 4% paraformaldehyde in PBS and permeabilized. Cells were incubated overnight at 4°C with anti-TIRC7 antibody (10µg/ml) or with anti-human IgG (hIgG, 5µg/ml, Sigma) as negative control. Cells were rinsed with PBS, and incubated with Cy3-conjugated goat anti-human IgG (1:250, Sigma) diluted in 5% milk/PBS. Cells were mounted with 50% (vol/vol) glycerol in PBS and confocal images were obtained by using Zeiss LSM 510 confocal laser scanning microscope.

### Statistical Analysis

Data analysis was performed using GraphPad Prism 8 software. The phenotypical differences between IR1 and IR1neg Tregs were assessed using the non-parametric Wilcoxon matched pairs signed rank test to compare percentage marker expression on IR1 and IR1neg from 72h IL-2 *in vitro* stimulated PBMCs from 8 healthy volunteers.

## Data Availability Statement

The raw data supporting the conclusions of this article will be made available by the authors, without undue reservation.

## Ethics Statement

The studies involving human participants were reviewed and approved by Ethical Comittee Charite. The ethics committee waived the requirement of written informed consent for participation.

## Author Contributions

All authors listed have made a substantial, direct, and intellectual contribution to the work, and approved it for publication.

## Funding

NU was supported by the BmBF German Grant Innovation for Individualized Medicine and by the European Union Fund for Regional Development (EFRE) (100394224). TM is supported by the German Research Foundation Grants MU 2864/1-3 and MU 2864/3-1.

## Conflict of Interest

The authors declare that the research was conducted in the absence of any commercial or financial relationships that could be construed as a potential conflict of interest.

## Publisher’s Note

All claims expressed in this article are solely those of the authors and do not necessarily represent those of their affiliated organizations, or those of the publisher, the editors and the reviewers. Any product that may be evaluated in this article, or claim that may be made by its manufacturer, is not guaranteed or endorsed by the publisher.

## Supplementary Material

Supplementary Figure 1.1 Gating strategy directly after isolation (t0). PBMC were purified to exclude debris (**Supplementary Figure 1.1A**). Doublets were discriminated and out gated (1.1B), Dead cells were excluded (**Supplementary Figure 1.1C**), CD3+, CD4+ T cells were identified (**Supplementary Figure 1.1D**). CD4+ T cells expressing high levels of CD25 with low expression of CD127 were considered Treg cells (**Supplementary Figure 1.1E**). Surface TIRC7 expression based on TIRC7 FMO control (blue) was measured (**Supplementary Figure 1.1F**)

Supplementary Figure 1.2 Gating after 72h IL-2 incubation. Debris, doublets and dead cells were excluded (like shown in **Supplementary 1.1A**–**C**). **(A)**. shows activated T-cells, cells expressing CD4 and medium to high (bright) levels of CD25, **(B)**. FOXP3 expressing activated T cells were gated and considered as FOXP3 Treg. **(C)**. TIRC7 expression on FOXP3 Treg was assessed and TIRC7+ Treg identified as IR1+ and TIRC7- as IR1neg. The blue histogram represents the TIRC7 FMO control and the red one the TIRC7 Panel. Marker expression on both subsets was measured for CTLA-4, CD62L, HLA-DR, GARP, IL-10, LAP, CD39, CD45RO, ICOS, Helios and CCR7.

Supplementary Figure 1.3 Living cells were selected as shown in **Supplementary Figures 1.1A**–**C**. Tregs were identified as CD4+ **(A)**, CD25+ CD127lo expressing **(B)** and FOXP3+ cells **(C)**. TIRC7 is used to separate IR1+ (TIRC7+ Treg) and Non-IR1 Tregs **(D)**. TIRC7+ Treg (IR1+) express high levels of FOXP3 at t0. After *in vitro*-stimulation with IL-2 (1,000UI/ml) for 72h, IR1 are induced and FOXP3 phenotype maintained.

Supplementary Figure 1.4 Gating strategy for non-activated CD45RA+ Tregs. Tregs were identified as CD4+ CD25+ CD127lo FOXP3+ cells isolated from PBMC. TIRC7 is added to identify IR1 cells (TIRC7+ Treg). CD45RA was used to analyze CD45RAhigh and CD45RAlo subset of Tregs. Shown is one representative example from 5 healthy donors. Marker expression is measured on the y-axis versus side scatter (FSC-A) on the x-axis.

Supplementary Figure 2 Overlaid histograms for marker expression. **(A)** Shows overlaid histograms with marker expression on IR1 (red) in comparison to IR1neg (blue) Treg after 72h IL-2 incubation (8 healthy volunteers). **(B)** Shows the mean fluorescent intensity of markers measured on Treg after 72h IL-2 incubation (8 healthy donors). The percent expression is shown by marker for IR1 (dark blue) versus IR1neg (light blue) Treg cells.

Supplementary Figure 3 Specificity of TIRC7 antibody. **(A)** TIRC7 antibody detected specifically recombinant TIRC7 protein only in COS7 transfected cells. **(B)**. TIRC7 protein was not detected in TIRC7 knockout mice (-/-) splenocytes in comparison to wild type (+/+) using TIRC7 mAb. **(C)** Biacore SPR analysis performed in varying concentrations to determine the kinetic constants or absorption effects as shown for mAb1.
