# Supplementary material for: Immune Regulatory 1 Cells: A Novel and Potent Subset of Human T Regulatory Cells
Source: Front Immunol. 2022 Feb 8;12:790775. doi: 10.3389/fimmu.2021.790775 (PMC8867398; doi:10.3389/fimmu.2021.790775)

Supplementary Figures

Figure 1.1

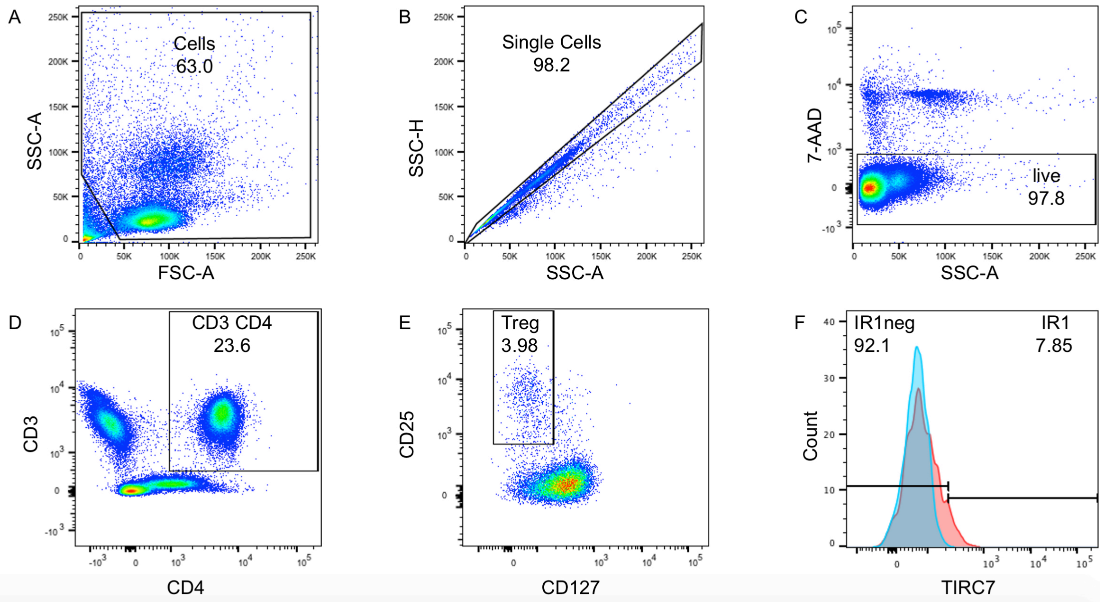

Supplementary Figure 1.2.

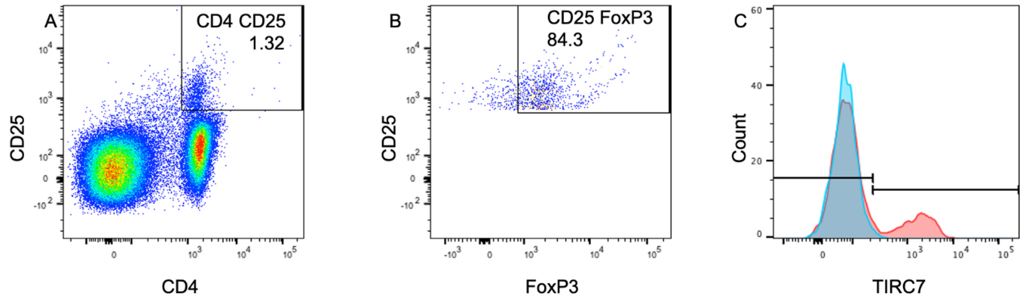

Supplementary Figure 1.3.

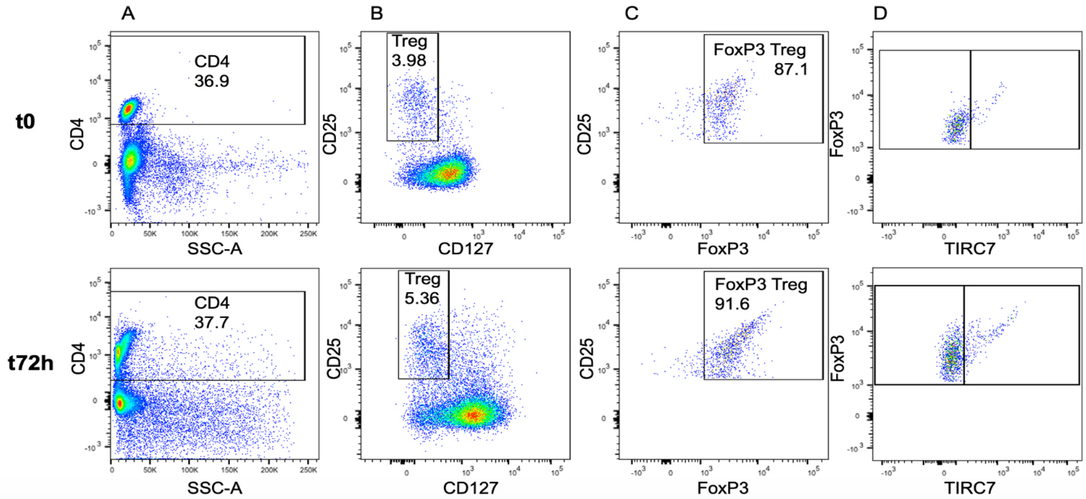

Supplementary Figure 1.4.

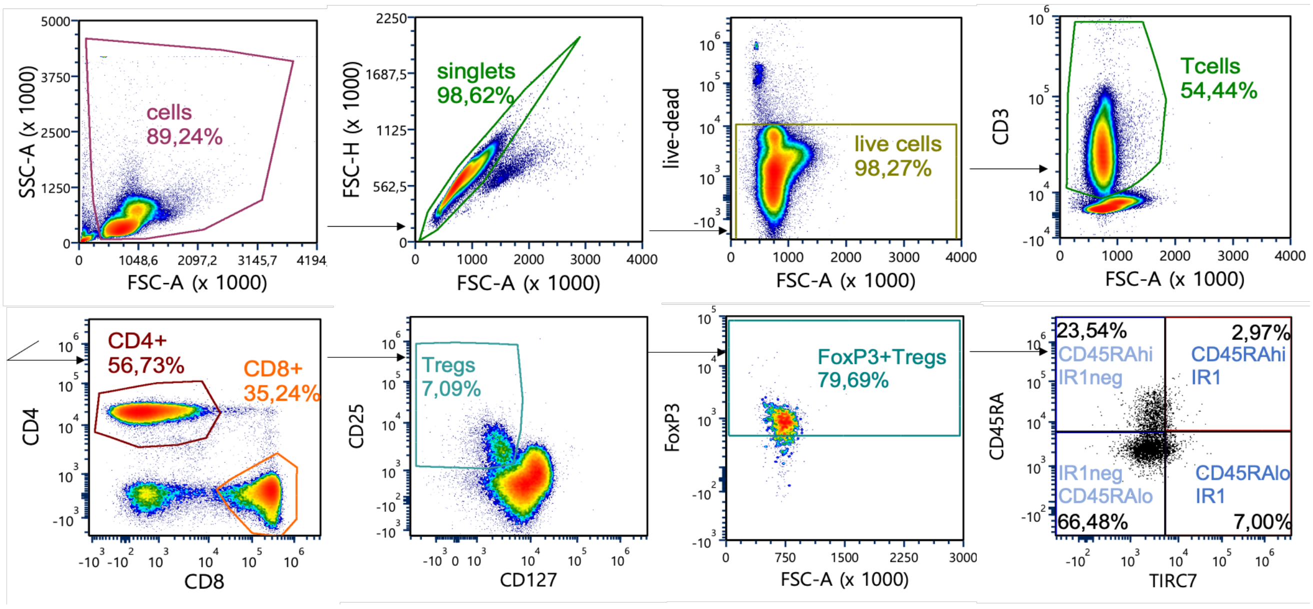

Supplementary Figure 2A

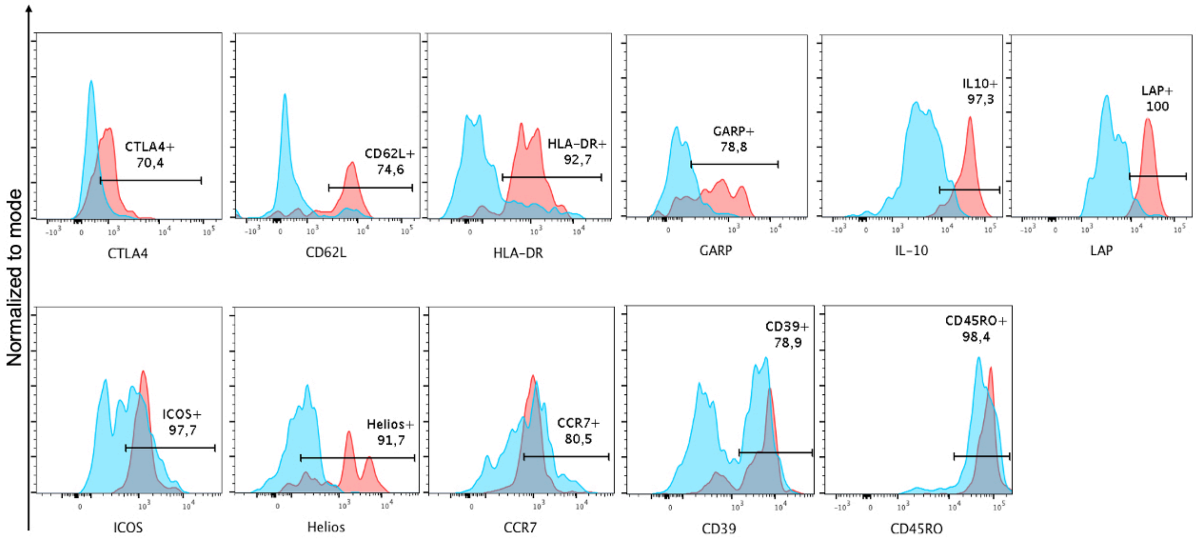

Supplementary Figure 2B

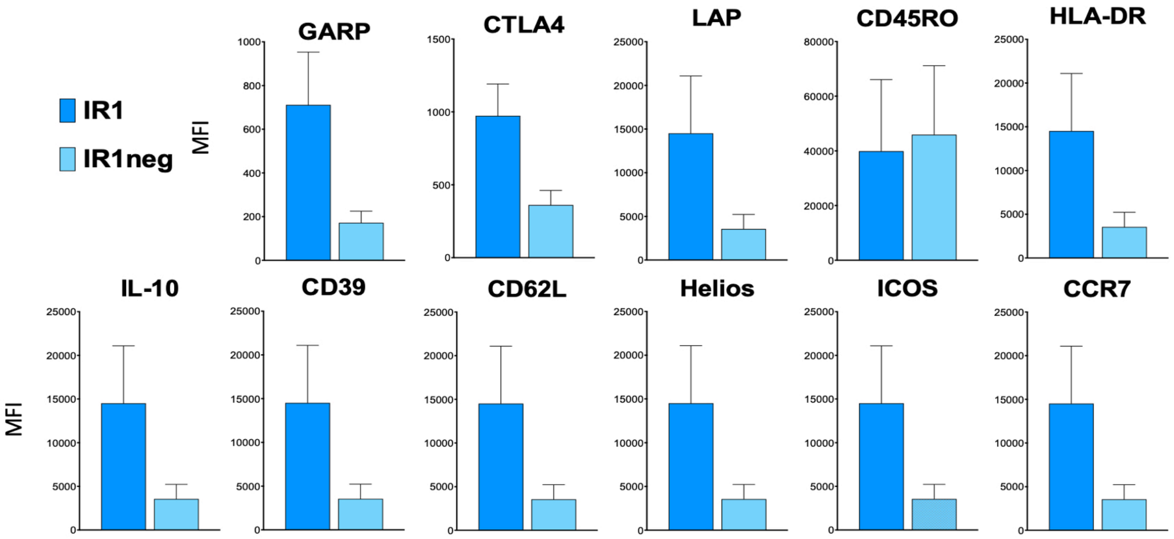

Supplementary Figure 3

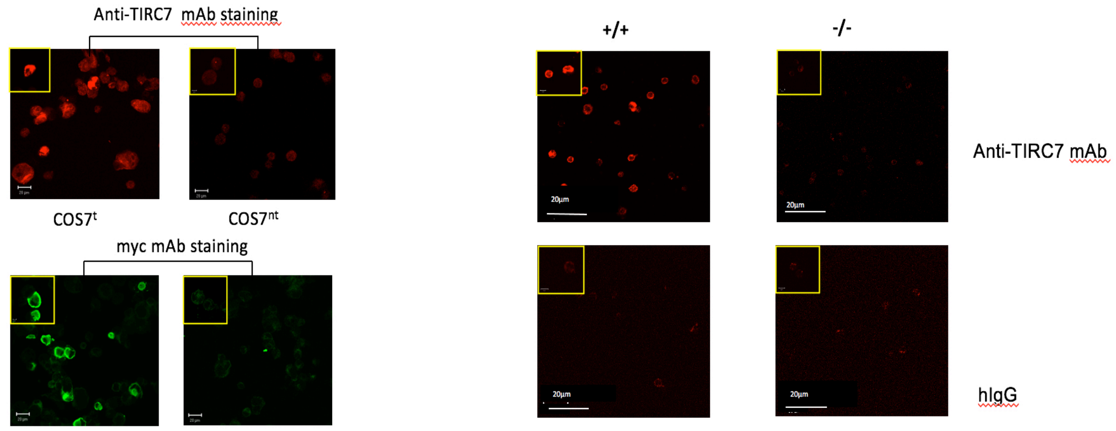

Supplement: Supplementary Figure 1.1 — Gating strategy directly after isolation (t0). PBMC were purified to exclude debris ( Supplementary Figure 1.1A ). Doublets were discriminated and out gated (1.1B), Dead cells were excluded ( Supplementary Figure 1.1C ), CD3+, CD4+ T cells were identified ( Supplementary Figure 1.1D ). CD4+ T cells expressing high levels of CD25 with low expression of CD127 were considered Treg cells ( Supplementary Figure 1.1E ). Surface TIRC7 expression based on TIRC7 FMO control (blue) was measured ( Supplementary Figure 1.1F ) [file Image_1.pdf]
